# Transcriptional responses of *Aedes aegypti* chemosensory tissues in response to volatile DEET and citronella oil

**DOI:** 10.1111/imb.70049

**Published:** 2026-06-04

**Authors:** Ivan Drahun, Jessica M. Sparrow, Cody W. Koloski, Bryan J. Cassone

**Affiliations:** ^1^ Department of Biology Brandon University Brandon Manitoba Canada; ^2^ Department of Veterinary Microbiology Western College of Veterinary Medicine, University of Saskatchewan Saskatoon Saskatchewan Canada; ^3^ Present address: Department of Biological Sciences Brock University St. Catharines Ontario Canada

**Keywords:** carbon dioxide, chemosensation, gustatory receptors, olfaction, physiology, repellents

## Abstract

DEET and citronella oil are two of the most widely used mosquito repellents; however, their impact on chemosensory tissues is not fully understood. In their vapour phase, the repellency effect involves the olfactory system, with mosquitoes detecting and responding to diverse odorants through their antennae and mouthparts (maxillary palps and proboscis). In this study, we first performed repellency behavioural assays that indicated both DEET and citronella effectively impaired female *Aedes aegypti* host‐seeking capabilities (i.e. landing on a host and taking a blood meal). We next used RNA sequencing to assess global gene expression changes in *Ae. aegypti* antennae, mouthparts and headless bodies in response to volatile repellent exposure. The resultant transcriptional changes were considerably greater in the mosquito mouthparts than in the other tissues. The most notable responses in olfactory‐related genes to both repellents were the suppressed expression of the three subunits comprising the mosquito CO_2_ receptor (Gr1, 2 and 3). Genes associated with mitochondrial function and metabolism were also suppressed. Exposure to DEET was further characterized by substantial inhibition of cytochrome P450 mRNA expression, the primary detoxification enzymes in insects. Overall, our study provides novel insights into the olfactory and physiological responses of *Ae. aegypti* chemosensory tissues in response to DEET and citronella exposure.

## Graphical abstract

- *Aedes aegypti* females were significantly repelled by both volatile DEET and citronella oil in comparison to control (ethanol‐exposed) mosquitoes.
- Global gene expression changes were largest in the mosquito mouthparts, with notable downregulation of the three subunits comprising the mosquito CO_2_ receptor (Gr1, 2 and 3).
- Both repellents produced transcriptional responses indicative of mitochondrial dysfunction and reduced metabolism. DEET exposure also caused rampant cytochrome P450 inhibition.

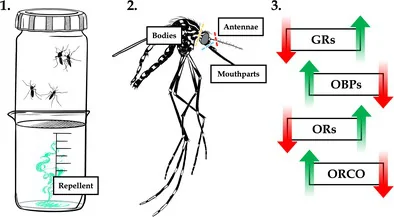

## INTRODUCTION

Mosquitoes (Diptera: Culicidae) use their olfactory system to detect and respond to spatial and volatile repellents (Afify et al., 2019; Ray, 2015). They possess three chemosensory appendages capable of sensing a plethora of odours from their surrounding environment: antennae, maxillary palps and labella (tip of proboscis) (Carey & Carlson, 2011; Potter, 2014; Saveer et al., 2018). Each of these appendages is covered in sensory hairs (sensilla), which typically contain 2–5 olfactory sensory neurons (OSNs) per sensillum (McIver, 1982). As the dendrites of OSNs are located within the aqueous sensillar lymph, odorant molecules must first enter the structure through various pores and bind to an odorant‐binding protein (OBPs) within the lymph (Figure S1). Once bound, the OBP helps solubilize the odorant molecule and transport it to chemoreceptors embedded on the surface of OSNs (Pask & Ray, 2016; Takken, 2000). There are three main types of chemoreceptors used in mosquito olfaction: odorant receptors (ORs), ionotropic receptors (IRs) and gustatory receptors (GRs) (Leal et al., 2013; Nakagawa & Vosshall, 2009; Suh et al., 2014). When an odorant molecule binds to a specific chemoreceptor, it triggers a cascade of events that leads to the generation of an electrical signal that travels via the OSN axon to a specific glomerulus in the brain to be interpreted as a particular odour (Wheelwright et al., 2021). Odorant‐degrading enzymes (e.g. cytochrome P450s, glutathione S‐transferases) maintain olfactory system sensitivity by rapidly removing odorant molecules from the vicinity of ORs (Durand et al., 2011; Lu et al., 2021). The underlying molecular mechanism(s) by which most repellents affect the mosquito chemosensory tissues, including potential xenobiotic and other physiological responses, are not fully understood.

N,N‐diethyl‐3‐methylbenzamide (DEET) has been commercially available for over 65 years and remains the ‘gold standard’ of mosquito repellents (Fradin, 1998; Pickett et al., 2008). Applied topically to the skin, DEET provides several hours of protection from mosquitoes and other haematophagous insects (Fradin & Day, 2002). It is clear that DEET in its vapour phase alters mosquito olfactory responses, but its mode of action is not yet resolved (Afify et al., 2019; DeGennaro, 2015). Studies from *Aedes* (DeGennaro et al., 2013) and *Culex* (Xu et al., 2014) mosquitoes indicate that DEET directly functions through the odorant receptor co‐receptor (Orco)/OR pathway. There are currently three main hypotheses as to how DEET affects mosquitoes: (1) ‘confuses’ by modulating sensitivity of ORs in association with host odours to effectively scramble the insect odour code (DeGennaro, 2015; Jaramillo Ramirez et al., 2012; Pellegrino et al., 2011); (2) ‘smell and avoid’ where DEET activates specific ORs and potentially other chemoreceptors to be perceived as a harmful/adverse odour by the mosquito (Bohbot et al., 2011; Bohbot & Dickens, 2010; Stanczyk et al., 2010; Syed & Leal, 2008; Xu et al., 2014); and (3) ‘masking’ where DEET decreases the volatility of attractive odorants, thereby resulting in overall reduced OR responses to host‐seeking attractants (Afify et al., 2019; Syed & Leal, 2008). It is conceivable that volatile DEET may have more than one mode of action, and also appears to be multi‐modal with documented contact chemorepellency (DeGennaro et al., 2013; Dennis et al., 2019).

A variety of plant‐derived agents represent alternative mosquito repellents to DEET. Citronella oil, derived from the leaves and stems of various *Cymbopogon* (lemongrass) species, is one of the most commonly used. The main constituents of the essential oil are the monoterpene aldehyde citronellal, with its associated oxidation products citronellol and geraniol (Subramanya et al., 2022). Other active compounds in citronella oil with insect repellent properties include camphor, eucalyptol, eugenol, linalool and citral (Moore et al., 2006). A meta‐analysis indicated that the essential oil in its vapour phase confers strong repellency to *Aedes*, *Anophele*s and *Culex* mosquitoes, which is comparable to DEET in the short term (Kongkaew et al., 2011). Limited research has been done to investigate the mode of action of citronella oil, but given its strong, distinctive scent, it may function similarly to the masking hypothesis of DEET. Kwon et al. (2010) demonstrated that volatile citronellal directly activates the TRPA1 gene in *Anopheles*, which in turn may induce antennal OSNs to detect odorants and suppress host‐seeking behaviours. Further, simulated molecular docking analyses indicated that OBP4 binds citronellal and transports it to AgamORC7 in *An. gambiae* s.s. (Wu et al., 2020). It is conceivable that the other components of citronella oil with insect repellent properties could antagonize different molecular targets in the mosquito olfactory pathways, leading to synergistic or amplifying effects.

*Aedes aegypti* is a highly anthropophilic and endophilic species with a broad distribution globally, particularly in tropical and subtropical regions (Gómez et al., 2022). It is a primary vector of several pathogenic viruses of public health concern, including yellow fever, dengue, chikungunya and Zika (Laporta et al., 2023). In addition to vaccines (where available), repellents represent an important preemptive line of defence against mosquito‐transmitted diseases. However, extensive use of DEET and other repellents formulated from one or a small number of active ingredients could rapidly select for ‘insensitive mosquitoes’ in natural populations. This concern is warranted, as variation in the sensitivity to DEET has been previously reported in mosquito populations (Frances et al., 2004; Ruthledge et al., 1978; Stanczyk et al., 2010). Above and beyond elucidating their impacts to insect olfaction and physiology, the identification of specific molecular targets (e.g. GRs, OBPs, ORs) may advance research towards the development and formulation of novel repellents that minimize the chances of insensitivity spreading in natural populations.

The overall goal of our study was to identify putative molecular targets associated with DEET and citronella oil repellency in mosquitoes. To do this, we first carried out behavioural assays via artificial membrane blood feeding to characterize the repellency efficacy of both repellents towards *Ae. aegypti* females (e.g. landing on skin and engorging in a blood meal). In addition, we exposed mosquitoes to each repellent in its vapour phase and harnessed RNA sequencing to catalogue the subsets of genes induced and suppressed within the mosquito chemosensory tissues (antennae, maxillary palps and proboscis) and bodies.

## MATERIALS AND METHODS

### Mosquito colony rearing and maintenance

Eggs of *Ae. aegypti* (COSTA RICA, MRA‐726, deposited by W.G. Brogdon) were obtained from the Malaria Research and Reference Reagent Resource Center (MR4) as part of the BEI Resources Repository, NIAID, NIH. Colonies were maintained in an environmental chamber with a condensate recirculator system (Model 7301‐75‐2, Caron, Marietta, OH, USA) under controlled conditions of 27°C, 80% RH and a 12 h/12 h light‐dark cycle with 1‐h crepuscular transitions. For each generation, eggs were placed in plastic trays (36 × 23 × 7 cm) containing 1 L of distilled water (changed every other day) at low density (~150 per tray) and fed daily (~1 g) pulverized Benefel Original dog food (Purina, St. Louis, MO, USA). Pupae were picked and transferred to 30.5 × 30.5 × 30.5 cm emergence cages (BioQuip, Rancho Dominguez, CA, USA). Upon emergence, adult mosquitoes were fed a 10% sucrose solution through cotton wicks. Only adult females (~3 days post‐eclosion) were used in the present study. Experimental individuals were subjected to a 24‐h starvation period prior to commencement of the respective assay.

### Repellency behaviour assays

Two treatments and a control were formulated for our experiments: 50% DEET, 50% citronella oil (Organic Harvest, Brooksville, FL, USA), or 50% ethanol (control). The concentrations were produced by diluting >98% N,N‐diethyl‐m‐toluamide (Tokyo Chemical Industry America, New Brunswick, NJ, USA) or 100% citronella essential oil in molecular grade absolute ethyl alcohol (ethanol) (Greenfeld Global, Toronto, ON, Canada). The control was diluted in ultra‐pure water (Bio Basic, Markham, ON, Canada).

Adult females (*n* = 15) were randomly assigned to a treatment/control and fed for 20 min on a feeding system with a collagen membrane (PS6120, Hemotek, Blackburn, United Kingdom) containing heparinized sheep blood (CL2581‐100H, Cedarlane, Burlington, ON, Canada) evenly covered with 1 mL of the given treatment. Ethanol‐soaked paper towels were used to clean the feeder between trials, and control and treated surfaces were dried for 30 min prior to use to allow for maximum vaporization (Dye‐Braumuller et al., 2017). Over the 20‐min time course, we recorded the number of individuals that (1) landed on the feeder, and (2) blood fed, with the latter validated by cataloguing the number of engorged individuals. Each control and treatment exposure was performed independently on cages of mosquitoes (i.e. no‐choice experiment). These experiments were replicated 5 times for a total of 75 mosquitoes per control/treatment. Statistical differences were assessed by Kruskal‐Wallis non‐parametric H tests followed by Dunn's test post hoc test (*p* < 0.05), using the MultNonParam and dunn. test packages in R (R Core Team, 2021).

### Transcriptional responses of *Ae. Aegypti* to volatile repellent exposure

#### Experimental design

**FIGURE 1:**
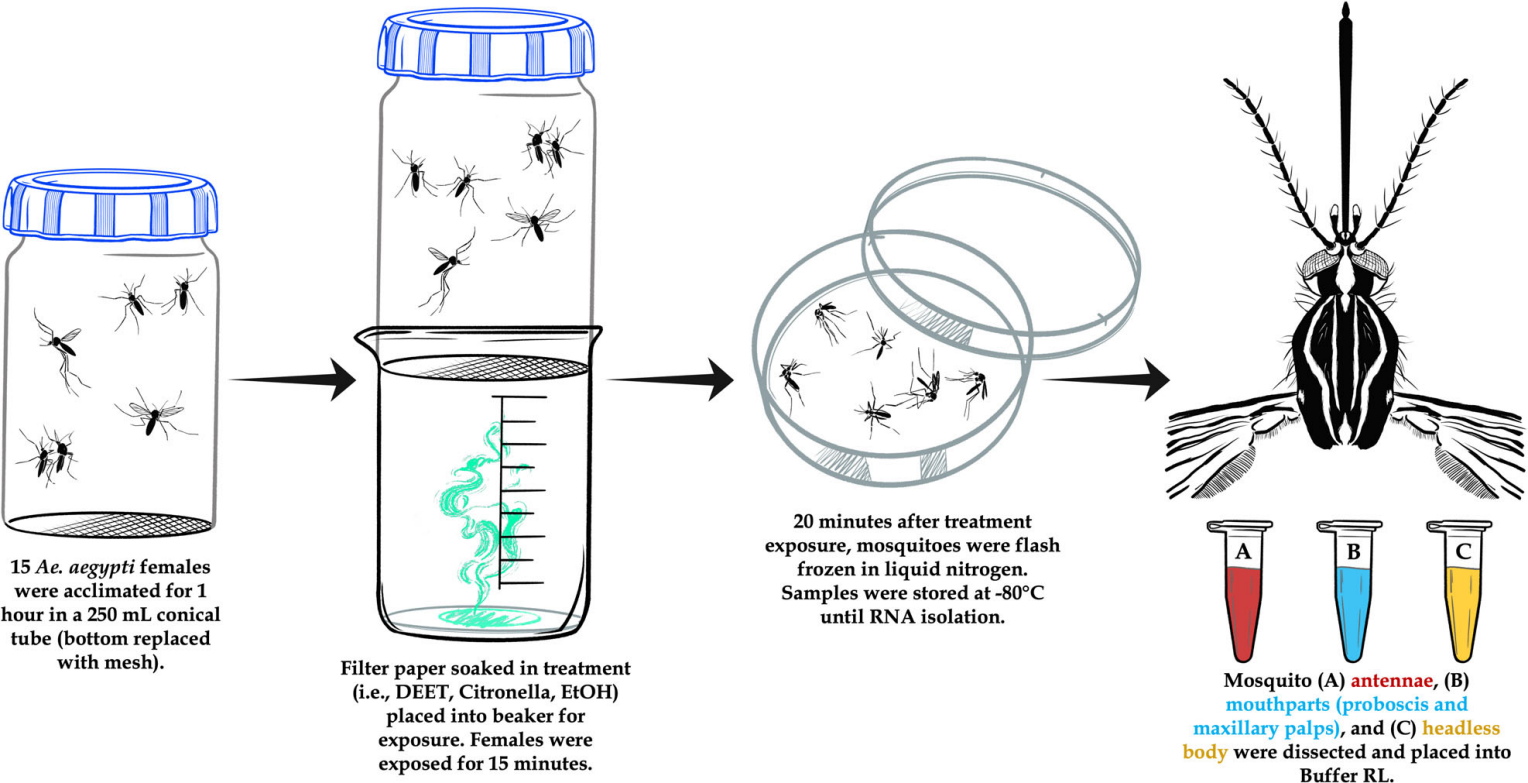
Overview of the experimental design used for mosquito exposure to treatments (DEET, citronella, or ethanol control) and sample collection. (a) *Aedes aegypti* females were acclimated for 1 h in a 250 mL conical tube (bottom replaced with mesh); (b) filter paper soaked in treatment was placed into beakers for a 15 min volatile exposure; (c) 20 min post‐exposure, mosquitoes were flash‐frozen; (d) antennae, mouthparts (proboscis and maxillary palps) and headless bodies were dissected for RNA extraction.

To negate competing odours, these experiments were conducted in closed laboratories with both the control and treated trials performed in different facilities to ensure no cross‐contamination. Further, experimental individuals were taken from maintenance cages that were closed off from the trials. Adult females were randomly assigned to one of three treatments: 50% DEET, 50% citronella oil, or 50% ethanol/control (see above for the preparations). Figure 1 displays the experimental setup for the repellent exposures, including our custom vessels used for each trial. Mosquitoes were acclimated at room temperature for 1 h in 250 mL (6 × 16 cm) custom vessels (20 females per tube). Each vessel consisted of a conical centrifuge tube fitted with mesh fashioned onto an opened terminal end and a holed‐out inverted plastic cup of the same diameter stacked directly on top of the tube (holed‐out position adjacent to mesh), sealed together using adhesives. At the commencement of each trial, we placed the tube‐cup directly on top of an identical, lidless 250 mL tube containing 31.8 cm^2^ semidiscs of Whatman no. 1 filter paper saturated with 1 mL of DEET, citronella, or ethanol and dried for 30 min. Our experimental design ensured only volatile exposure (travelling upwards) between the treated surface and mosquitoes (i.e. no contact). Mosquitoes were exposed to a given treatment for 15 min, followed by a 20 min post‐exposure period. Individuals were then flash frozen in liquid nitrogen and stored at −80°C until RNA isolation. These trials were performed using four exposure vessels (single‐use) per treatment/control (4 × 3 = 12 vessels), and replicated three (citronella) or four (DEET and control) times with mosquitoes from different cohorts.

#### RNA extraction and sequencing

Mosquito tissue collections were done under a dissection microscope with *Ae. aegypti* females placed in sterilized Petri dishes of the same treatment/replicate. Using sterilized scalpels and forceps for each tissue, we made an incision to remove the head from the body. Paired antennae were subsequently excised, followed by the maxillary palps and proboscis. Mosquitoes were dissected in groups of 2 to 4, with the individual tissues placed in 1.5 mL microcentrifuge tubes containing buffer RL (Norgen Biotek Corp., Thorold, ON, Canada) that were on dry ice. Due to the microscopic nature of mosquito appendages, different amounts of each tissue were pooled per sample: 75 pairs of antennae, 50 sets of maxillary palps and proboscis and 25 headless bodies provided sufficient RNA concentration and quality for sequencing.

For each pooled replicate, total RNA was isolated using the Animal Tissue RNA Purification Kit (Norgen). We used the Nanophotometer NP80 to assess the RNA concentration (ng/μL), and sent the samples to the Génome Québec Innovation Centre (McGill University, Montreal, QC, Canada) for quality assessment via the BioAnalyzer 2100 (Agilent Technologies, Santa Clara, CA), mRNA stranded library (low input for antennae and maxillary palps and proboscis samples) preparation (New England Biolabs, Ipswich, MA, USA), and 100 bp paired‐end sequencing on the Illumina NovaSeq 6000. We performed bcl conversions and demultiplexing using CASAVA 1.8.2, and image deconvolution and quality value calculations via the Illumina GA pipeline (version 1.6). The raw sequence reads can be retrieved from the NCBI short sequence read archive (SRA) under the accession number PRJNA793033 (Cassone, 2026).

#### Differential expression analysis

Demultiplexed raw reads were imported into FASTQC (Andrews, 2010) to evaluate overall sequence quality. We used CLC Genomics Workbench v20.0.4 (Aarhus, Denmark) for adapter index trimming and removal of poly (A) tails (ambiguous limit = 2, quality limit = 0.05, ambiguous limit = 2). Preprocessed reads were mapped to the unspliced gene set of the most recent *Aedes aegypti* reference genome (19,804 genes), AaegL5 (Matthews et al., 2018) using the following optimized parameters: mismatch cost = 2, insertion cost = 3, deletion cost = 3, length fraction = 0.5 and similarity fraction = 0.9. Only reads that mapped uniquely (i.e. unambiguously) to one gene were included in the count data. Broken pairs were included, and paired‐end reads were counted as independent.

We imported the read count data into the DESeq2 package (Love et al., 2014) in RStudio v2025.05.0 (RStudio Team, 2019) to identify genes differentially expressed between the control and each repellent (DEET and citronella). The median‐to‐ratio method was used to estimate normalization factors for sequencing depth, and similarities in global expression profiles among samples were explored by principal components analysis. We fitted the dispersion parameters and coefficients (e.g. log2 fold changes) to a negative binomial distribution, with the values for each gene estimated using the approximate posterior estimation for GLM (apeglm) shrinkage method. Significance was assessed via Wald tests, with differentially expressed genes having *p* < 0.05 and log_2_ fold changes less/greater than 0.58 (i.e. fold change = 1.5). As a small amount of cross‐contamination between chemosensory tissues can cause considerable aberrance in gene expression profiles (Rinker et al., 2015), we only included significant genes with mean expression values >100 that were expressed at higher levels in the given chemosensory tissue (e.g. higher in maxillary palps and proboscis than antennae or vice versa) for DEET/control or citronella/control. For each comparison, significant gene sets were functionally annotated using the Database for Annotation, Visualization and Integrated Discovery (DAVID 2021) with VectorBase gene IDs as the identifier (Huang et al., 2007; Huang et al., 2009). This included implementing the functional annotation clustering tool for enrichment of GO, INTERPRO, SMART and other annotation terms in the sublists. Significant clusters were defined as having threshold EASE enrichment scores ≥1.3 (equivalent to *p* ≤ 0.05) and ≥ 10 significant genes (default settings herein). Significant genes involved in olfaction were also catalogued, regardless of whether they were part of an enriched annotation cluster. This was achieved by comparing our candidate gene lists to olfactory genes (i.e. OBPs, chemoreceptors), derived from the AaegL5 database and extracted using BioMart in Ensembl Metazoa (Ye et al., 2022). The resultant list included 42 OBPs, 30 GRs, 72 ORs and 1 IR. Finally, we performed KEGG pathway analyses in DAVID (*p* < 0.05, ≥5 significant genes).

#### Quantitative real‐time PCR

Differential expression validation was performed on a subset of genes for the control and DEET comparison, using four samples per treatment. We generated cDNA from the maxillary palps and proboscis RNA using the GoScript Reverse Transcription System (Promega, Madison, WI, USA). We extracted the targeted mRNA sequences from VectorBase (Giraldo‐Calderón et al., 2015), and primer pairs were designed using Primer‐BLAST (Ye et al., 2012). Table S1 displays each of the primer pair sequences. The qPCR reactions (15 μL) were run in duplicate using QuantiNova SYBR Green master mix (Qiagen) and a Rotor‐Gene‐Q thermocycler (Qiagen). The thermal regime consisted of 95°C for 5 min, followed by 40 cycles of 95°C for 5 s and 56°C for 10 s. Cycle threshold (Ct) was determined using the Rotor‐Gene Q software (Qiagen). Standard curves were generated for each primer pair and efficiency was computed using QuantiNova Rotor‐Gene Q Series Software (Qiagen). Only primer pairs with sufficient efficiency values (1.00 ± 0.20) were used for further testing. Relative abundance was calculated by using the 2^−ΔΔCt^ method (Pfaffl, 2001; Schmittgen & Livak, 2008), using the *ACT* housekeeping gene as a reference (Dzaki et al., 2017). Significance was determined between treatments using one‐tailed independent *t*‐tests (*p* < 0.05).

## RESULTS

### Volatile DEET and citronella oil effectively repelled *Ae. Aegypti* mosquitoes

We first evaluated the effectiveness of both repellents to deter *Ae. aegypti* female host‐seeking behaviours. In total, 77% and 69% of individuals landed and blood fed on the control‐treated membrane feeder, respectively. Kruskal‐Wallis H tests indicated significant differences between the treatments and control in the number of mosquitoes that landed on the membrane feeder (DEET: 6.7%; citronella: 11%) (*χ* ^2^ (2) = 10.28, *p* = 0.006) and engorged on a blood meal (DEET: 0%; citronella: 2.7%) (*χ* ^2^ (2) = 11.67, *p* = 0.003). Pairwise post‐hoc analyses revealed that, compared to the control, significantly fewer mosquitoes landed on the membrane feeder treated with DEET (*Z* = 3.044, *p* = 0.002) and citronella (*Z* = 2.392, *p* = 0.017). Similarly, the number of *Ae. aegypti* taking a blood meal was significantly less for DEET (*Z* = 3.237, *p* = 0.001) and citronella (*Z* = 2.498, *p* = 0.012) in comparison to the control. No statistically significant differences were observed between DEET and citronella in the number of mosquitoes landing (*Z* = 2.759, *p* = 0.514) and blood feeding (*Z* = 2.602, *p* = 0.442).

### Global gene expression profiles are influenced by repellent exposure

**FIGURE 2:**
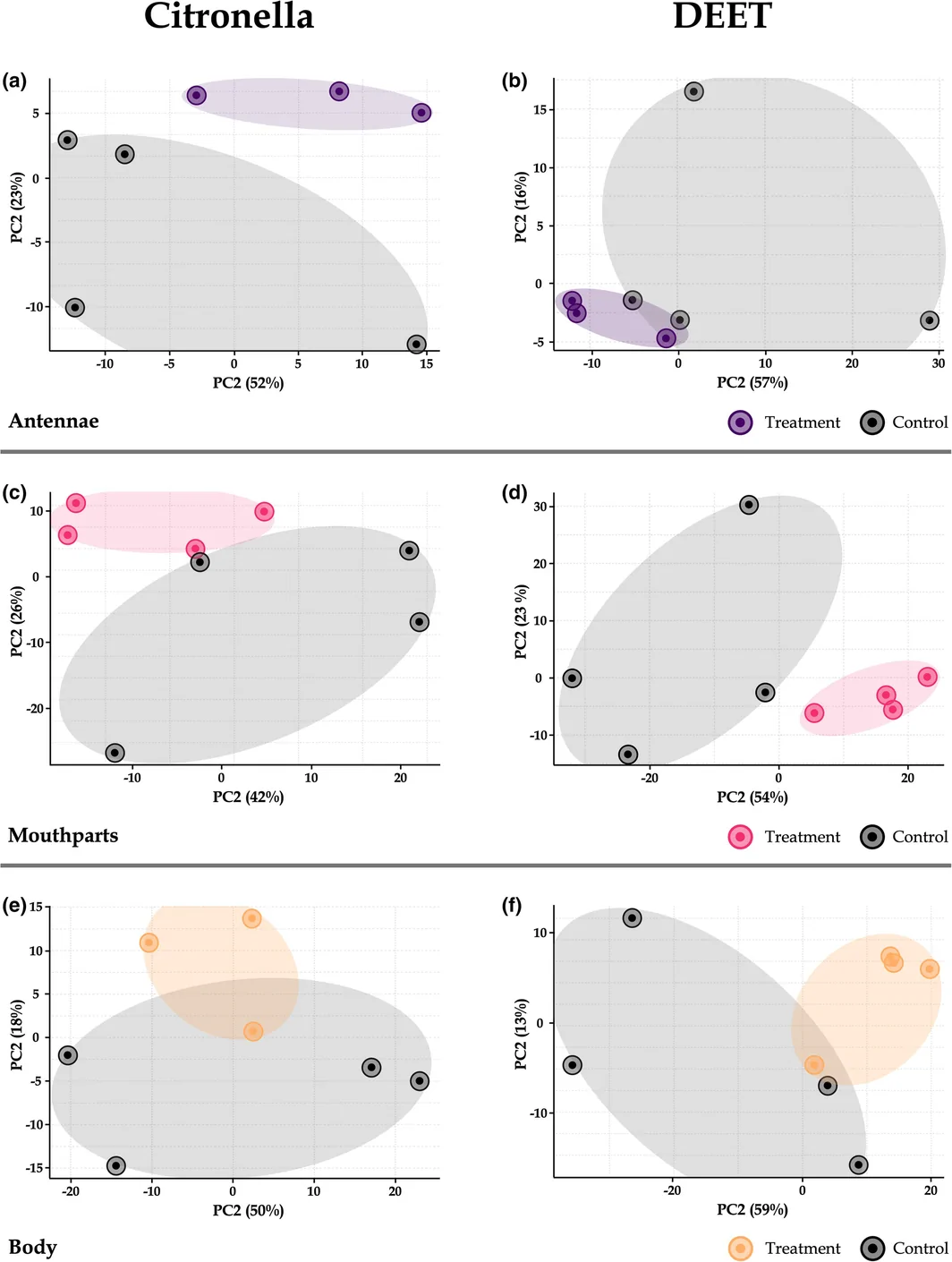
Principal component analysis (PCA) of cDNA libraries. These PCAs were constructed to compare global gene expression profiles between *Aedes aegypti* exposed to repellents (DEET or citronella) and the control (grey) for the antennae (purple), mouthparts (maxillary palps and proboscis) (red) and headless bodies (orange). The PCAs for the citronella and DEET comparisons are on the left and right, respectively.

We sequenced 33 cDNA libraries, with one DEET antennae replicate omitted due to an aberrant expression profile. This consisted of four replicates for each control and DEET treatment/tissue, and three replicates for each citronella treatment/tissue. In total, 2,318,812,680 raw reads of 100 bp were generated, with >80% of reads per sample mapping uniquely to the AaegL5 gene set (Table S2). The read counts for each gene/tissue/treatment are displayed in Table S3. Principal component analyses (PCA) were constructed to visualize the qualitative relationships among global expression profiles derived from control and DEET or citronella‐treated female mosquito antennae, palps and proboscis and headless bodies (Figure 2). Overall, the libraries tended to loosely cluster by their respective treatment, indicating that repellent exposure influenced the expression profiles of each *Ae. aegypti* tissue to some extent. The greater variability among control samples relative to those treated may be indicative of the latter inducing a more uniform, specific response (i.e. avoidance behaviour); whereas, control mosquitoes are not constrained by the repellent, allowing them to potentially exhibit a wider range of natural behaviours.

### Gene expression differences predominate in the maxillary palps and proboscis

For each tissue/treatment comparison, less than 7% of *Ae. aegypti* genes were differentially expressed. We identified the largest number of significant genes (i.e. *p* < 0.05 and fold change ≥1.5) in the maxillary palps and proboscis, with 894 and 432 for DEET and citronella, respectively. In contrast, the antennae exhibited minimal differential expression, with only 8 genes for DEET and 36 for citronella meeting our criteria for significance. There were a total of 353 significant genes for the bodies, 85% (301) of which were from the DEET treatment. Exposure to DEET resulted in suppression/downregulation of the majority of significant genes relative to the control in all three tissues: 97% (mouthparts), 75% (antennae) and 60% (bodies). This pattern was consistent for the mouthparts of citronella‐exposed mosquitoes (74%); however, a higher proportion of significant genes was induced/upregulated in the antennae (81%) and bodies (73%).

**FIGURE 3:**
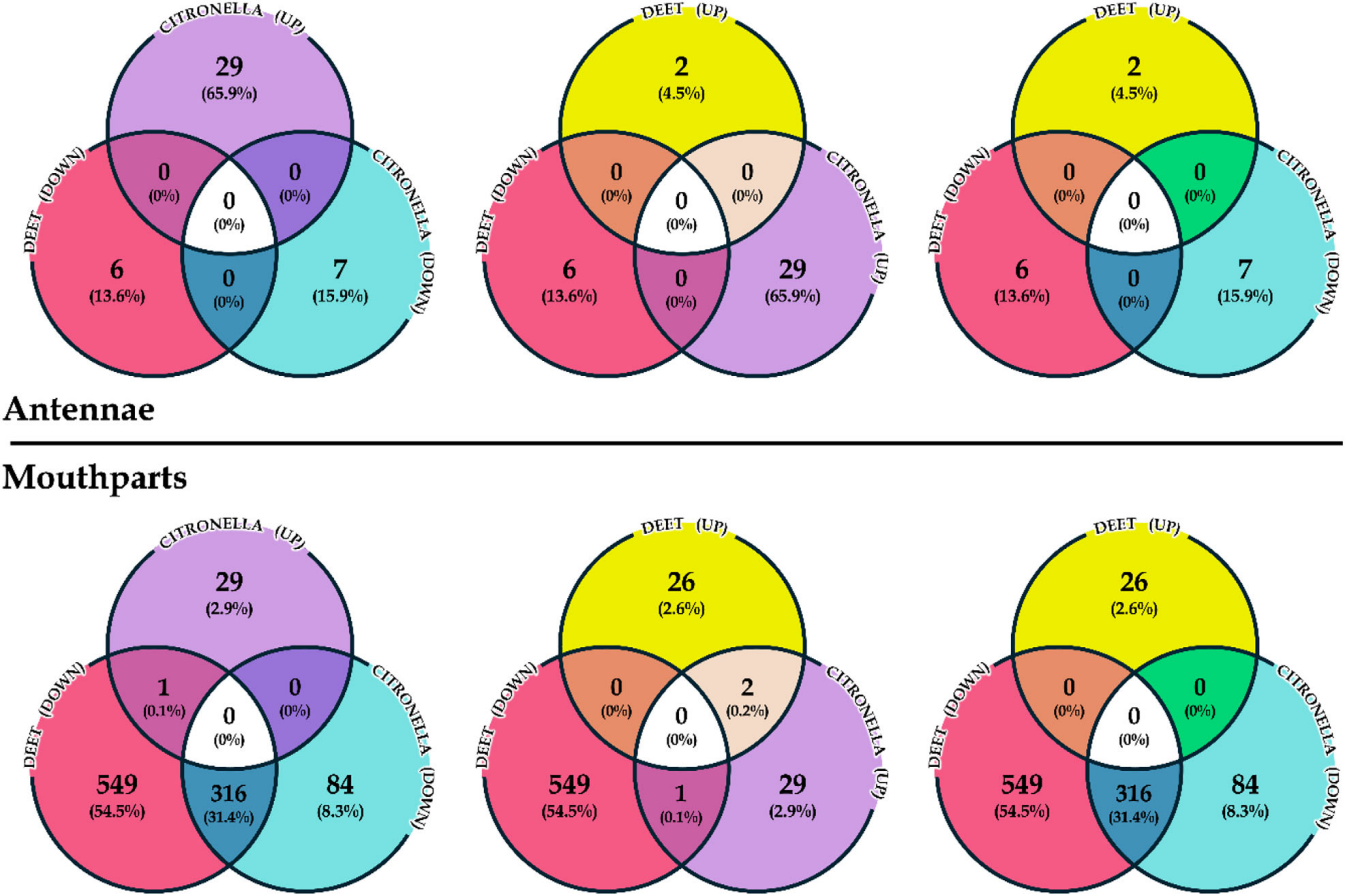
Venn diagrams displaying the partitioning of significant genes induced (upregulated) or suppressed (downregulated) due to volatile repellent (DEET or citronella) exposure in the antennae (up) and mouthparts (maxillary palps and proboscis) (down) relative to the control.

Figure 3 shows the partitioning of significant genes induced or suppressed due to repellent exposure in the mouthparts and antennae. With one exception, differences shared between repellent treatments showed the same direction of differential expression. We found that 79% of the subset of genes downregulated in the mouthparts due to citronella exposure were also downregulated in response to DEET. Only two genes were upregulated in response to both repellents, and there were no shared genes identified in the antennae datasets. Significant genes shared among tissue types were identified only between the mouthparts and bodies, totalling 54 genes (Figure S2).

### Volatile repellent exposure causes expression changes related to mitochondrial dysfunction, impaired metabolism and P450 inhibition

Tables 1 and S4 display the significant KEGG pathways and annotation clusters for each *Ae. aegypti* tissue, respectively. Both DEET and citronella exposure resulted in the downregulation of annotation clusters related to mitochondrial function in *Ae. aegypti* maxillary palps and proboscis, including oxidative phosphorylation, the tricarboxylic acid cycle, electron transport, ATP synthesis, lipoic acid metabolism, glycolysis and mitochondrial import/transport. Both repellents also suppressed cellular activities associated with the cytoskeleton (e.g. motor proteins and actin) and translation. KEGG pathways involved in metabolism were also suppressed, including aspects of amino acid, carbon, pyruvate, propanoate, glyoxylate and dicarboxylate metabolism. This downregulation of mitochondrial and metabolic processes was also identified in the bodies of DEET‐exposed mosquitoes, albeit not to the same extent. In addition, annotation clusters containing putative ODEs (cytochrome P450s/detoxification) and stress response genes (innate immunity and protein folding) were suppressed in the mouthparts due to DEET exposure. Transmembrane and transcription were the only annotation clusters induced in the mouthparts and/or bodies. No enriched annotation clusters or KEGG pathways were identified in the gene lists of the antennae.

**TABLE 1:** KEGG pathways showing significant downregulation of gene expression in *Aedes aegypti* female mouthparts and bodies in response to volatile DEET and citronella oil.

		Term	No. genes	*p*‐value
		Oxidative phosphorylation	62	3E‐36
		Metabolic pathways	179	1E‐27
		Carbon metabolism	40	5E‐19
		Citrate cycle (TCA cycle)	22	2E‐16
		Biosynthesis of amino acids	23	3E‐11
		2‐Oxocarboxylic acid metabolism	14	7E‐08
		Pyruvate metabolism	15	9E‐07
		Glycolysis/Gluconeogenesis	15	1E‐06
		Lipoic acid metabolism	10	1E‐06
	DEET	Glyoxylate and dicarboxylate metabolism	11	4E‐05
		Cytoskeleton in muscle cells	16	0.0003
		Cysteine and methionine metabolism	10	0.0004
		Pentose phosphate pathway	8	0.0005
		Glycine, serine and threonine metabolism	10	0.0009
		Bacterial invasion of epithelial cells	11	0.001
		Ribosome	23	0.0013
		Valine, leucine and isoleucine degradation	9	0.011
Mouthparts		Biosynthesis of nucleotide sugars	7	0.016
		Tyrosine metabolism	7	0.016
		Motor proteins	15	0.036
		Propanoate metabolism	6	0.048
		Oxidative phosphorylation	65	4E‐58
		Metabolic pathways	116	5E‐26
		Carbon metabolism	25	2E‐12
		Citrate cycle (TCA cycle)	15	1E‐11
		2‐Oxocarboxylic acid metabolism	11	2E‐07
	Citronella	Biosynthesis of amino acids	14	4E‐07
		Lipoic acid metabolism	7	5E‐05
		Ribosome	17	0.0004
		Glycolysis/Gluconeogenesis	8	0.0017
		Cytoskeleton in muscle cells	10	0.0031
		Glyoxylate and dicarboxylate metabolism	6	0.0076
		Propanoate metabolism	5	0.025
		Pyruvate metabolism	6	0.029
		Alanine, aspartate and glutamate metabolism	5	0.044
		Carbon metabolism	18	1E‐14
		Citrate cycle (TCA cycle)	12	2E‐13
		2‐Oxocarboxylic acid metabolism	10	1E‐10
		Metabolic pathways	38	3E‐08
	DEET	Biosynthesis of amino acids	10	6E‐08
Body		Glycolysis/Gluconeogenesis	8	2E‐06
		Lipoic acid metabolism	5	9E‐05
		Pyruvate metabolism	6	0.0003
		Glyoxylate and dicarboxylate metabolism	5	0.0008
		Cytoskeleton in muscle cells	6	0.0033

*Note*: The *p*‐value and number of significant genes are also displayed for each pathway/treatment/tissue. No significant KEGG pathways were identified in the antennae, and no KEGG pathways were upregulated in comparison to the control.

### Olfaction is modulated by repellent exposure

**FIGURE 4:**
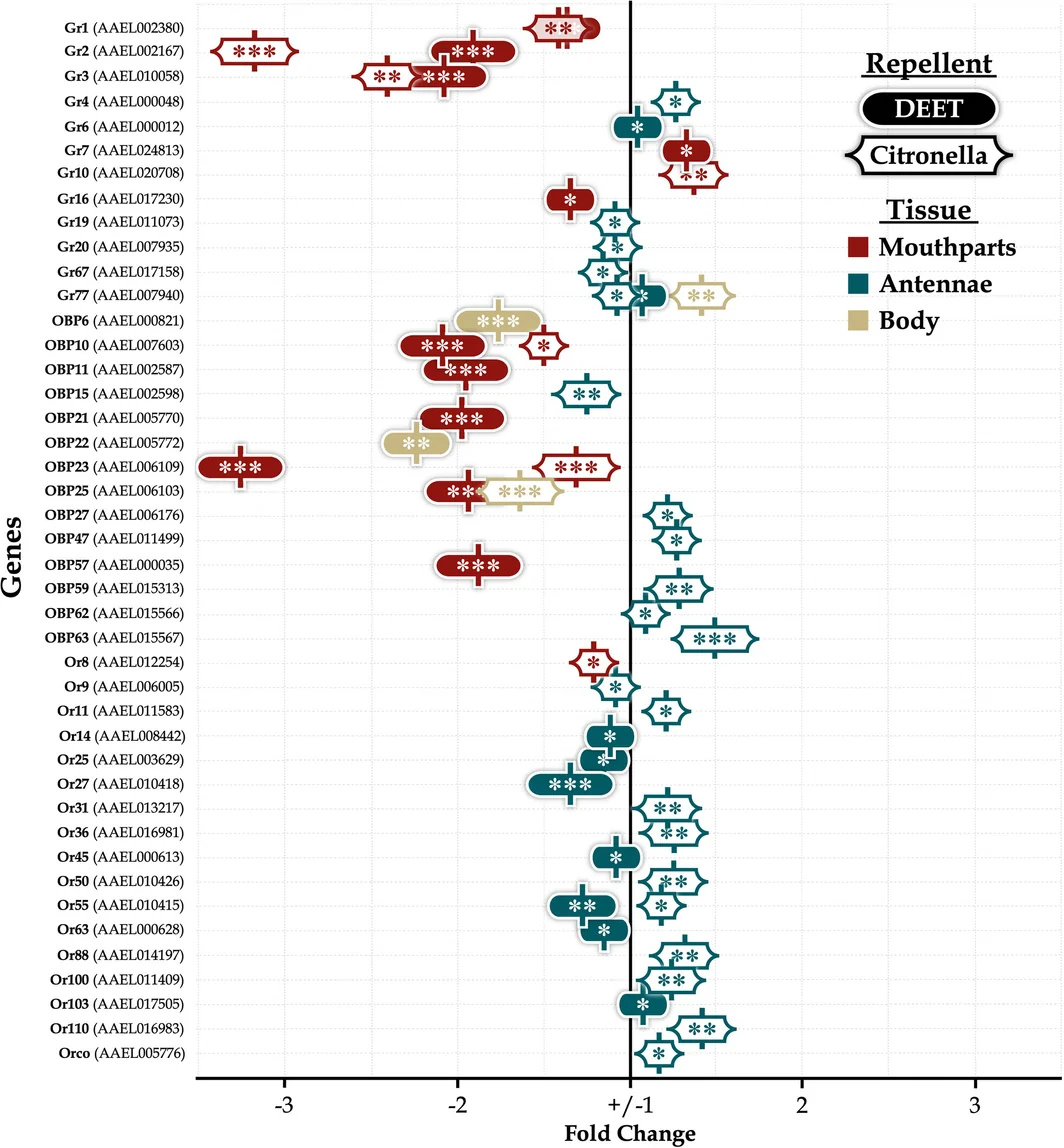
Differentially expressed (*p* < 0.05) OBPs and chemoreceptors (ORs and GRs) identified in *Aedes aegypti* mouthparts, antennae and bodies in response to volatile DEET or citronella exposure. Fold change differences relative to the control are displayed on the *X*‐axis.

We next searched for differential expression of key olfactory pathway genes in response to DEET or citronella regardless of enrichment. We used a less conservative *p* < 0.05 with no threshold for minimum fold‐change to ensure all putative candidate genes were identified, with the caveat that these gene lists are likely to contain more false positives. It must be emphasized that our analysis represents an underestimate of *Ae. aegypti* chemokine receptors, particularly IRs, as predicted through gene modelling (Matthews et al., 2018). Figure 4 shows the differentially expressed OBPs and chemoreceptors (ORs and GRs) identified in *Ae. aegypti* mouthparts, antennae and bodies in response to volatile DEET or citronella.

The antennae contained the largest number of significant olfactory genes, with 21 (6 OBPs, 9 ORs, 5 GRs, ORCO) and 9 (7 ORs and 2 GRs) for citronella and DEET, respectively. However, none of these genes were differentially expressed at fold changes >1.5, with the vast majority (87%) below 1.3. For the maxillary palps and proboscis, 11 (6 OBPs and 5 Grs) significant genes were identified for DEET and 7 (2 OBPs, 1 OR and 4 GRs) for citronella. Most notable were Gr1 (AAEL002380), Gr2 (AAEL002167), Gr3 (AAEL010058), Obp10 (AAEL007603) and Obp23 (AAEL006109); these genes were suppressed in response to both repellents, typically at fold changes >2 with *p* < 0.005. For the bodies, only four significant genes (3 OBPs and 1 GR) were identified and each was specific to a given repellent.

### Validation of differential expression by qPCR

All four genes tested showed significant differential expression between the DEET and control samples, with the orders of magnitude in line with the RNA sequencing results. DEET exposure resulted in downregulation in the expression of Gr2 (2.15‐fold), Gr3 (1.92‐fold), Obp10 (2.3‐fold) and Obp23 (3.06‐fold), with all pairwise comparisons showing a threshold for significance of *p* < 0.001.

## DISCUSSION

Mosquitoes detect, process and ultimately respond to volatile and spatial repellents through their highly sophisticated and sensitive olfactory systems; however, the precise mechanism(s) of action of most repellents is not fully understood (Afify & Potter, 2020; Clark & Ray, 2016; DeGennaro, 2015). DEET and citronella oil are among the most commonly used repellents worldwide, and our results indicate that in their vapour phase, both effectively deter *Ae. aegypti* females from landing on a host and taking a blood meal. Exposure to volatile DEET and citronella provoked greater transcriptional responses in the mosquito mouthparts (maxillary palps and labella) than in the antennae and bodies. This was a particularly surprising finding, as the *Ae. aegypti* antennae are the primary olfactory appendages, housing a significantly larger number and greater diversity of sensilla compared to the maxillary palps and labella (Herre et al., 2022; Hill & Smith, 1999; Steward & Atwood, 1963; Sutcliffe, 1994). Nonetheless, the mouthparts play key roles in host detection (Bohbot et al., 2014; Sparks et al., 2014), and the palps contain OSNs documented to detect and respond to specific repellent compounds (Shii et al., 2021; Syed et al., 2011). Overall, we identified notable similarities in the gene expression profiles of *Ae. aegypti* exposed to DEET or citronella; however, each repellent also showed distinct molecular signatures.

Our most intriguing finding was the downregulation of Grs 1, 2 and 3 in the mouthparts at relatively high fold changes in response to both DEET and citronella exposure. These three Grs are the subunits that constitute the carbon dioxide (CO_2_) receptor of *Ae. aegypti* and other mosquitoes, with Gr1 and Gr3 forming the core receptor and Gr2 likely modulating receptor sensitivity (Erdelyan et al., 2012; Kumar et al., 2020). Consistent with our findings, these GRs are expressed in specialized OSNs (cpA) housed in sensilla on the maxillary palps (Bohbot et al., 2014; Grant & O'Connell, 2007; Lu et al., 2007; Syed & Leal, 2007). In addition to being the primary host‐seeking attractant for mosquitoes (Turner et al., 2011), CO_2_ also serves as a behavioural activator by enhancing mosquito sensitivity to other host cues, such as human skin odour (Dekker et al., 2005; Healy & Copland, 2000), heat (Liu & Vosshall, 2019; McMeniman et al., 2014) and visual cues (Vinauger et al., 2019). Moreover, the OSNs that detect airborne CO_2_ also detect other compounds, many of which are found in human skin odour (Lu et al., 2007; Tauxe et al., 2013; Turner et al., 2011; Turner & Ray, 2009). Therefore, assuming the downregulation of Gr1, 2 and 3 mRNA expression results in impairment of Gr function, exposure to DEET and citronella may lead to broad‐ranging dysfunction in mosquito sensory behaviours (i.e. host‐seeking). The specific *Aedes* OBPs involved in CO_2_ binding and transport are not known, but Obp10 and Obp23 share similar patterns of downregulation to the GRs in the mouthparts and; thus, represent potential candidates. Both of these OBPs may play diverse pleiotropic roles in *Ae. aegypti*, including host‐seeking, blood feeding, reproduction and disease transmission (Guo et al., 2019; Sim et al., 2012; Dong et al., 2021). It is also conceivable that cpA can respond to CO_2_ in the absence of OBPs, as evidenced by some *Drosophila* olfactory sensilla (Larter et al., 2016; Xiao et al., 2019).

In addition to olfactory responses, *Ae. aegypti* exposed to volatile DEET appeared to activate xenobiotic responses. We identified a robust downregulation of cytochrome P450s in the mouthparts, consistent with previous toxicological and biochemical studies on mosquitoes exposed to the repellent (Bonnet et al., 2009; Corbel et al., 2009; Jaramillo Ramirez et al., 2012). Moreover, P450 inhibition has been observed in other arthropod taxa (Koloski et al., 2019; Koloski & Cassone, 2022), and in response to both short (1 min) and prolonged exposures to sub‐lethal concentrations of different odours (Jaramillo Ramirez et al., 2012; Mappin et al., 2023). Insects have evolved a multi‐phase system to survive exposure to natural toxins and synthetic insecticides, including reduced cuticle penetration, enhanced metabolic detoxification and increased excretion (Scott & Wen, 2001; Wu & Hoy, 2016). In insects, P450s are responsible for catalysing the oxidation, reduction, or hydrolysis of xenobiotics. The observed inhibition of these enzymes may lead to accumulation of the unmetabolized chemical, though any resultant impact on the insect (e.g. fitness) was not apparent from our study and would require further research. Downregulation of P450s was less extensive in the mouthparts of mosquitoes exposed to citronella, though 19 of these genes were inhibited. Other key xenobiotic‐associated gene families (e.g. insect cuticle proteins, glucuronosyltransferases, glutathione S‐transferase, carboxyl/cholinesterases) showed gene expression changes to some extent, but were not significantly enriched as clusters.

Exposure to both volatile DEET and citronella also produced transcriptional changes suggestive of mitochondrial dysfunction and impaired metabolism in *Ae aegypti* mouthparts and, to a lesser extent, bodies. DEET has been linked to mitochondrial dysfunction in *Caenorhabditis elegans* (Shin et al., 2024) and humans (Delic et al., 2021), and citronella may attenuate oxidative stress‐induced mitochondrial damage (Yin et al., 2022). Similar impacts on vertebrate mitochondrial function have been documented after exposure to a variety of insecticides (Guven et al., 2018; Miao et al., 2022; Taha et al., 2021; Taha et al., 2022; Wei et al., 2024; Xu et al., 2022), but little research has been undertaken for insects. However, certain odours (e.g. 1‐octen‐3‐ol) have been shown to induce significant mitochondrial dysfunction in *Drosophila* (Macedo et al., 2020). Moreover, there is some evidence from 2D‐PAGE and MALDI‐TOF mass spectrometry that DEET affects proteins related to synapses and ATP production in *Ae. aegypti* (Portilla Pulido et al., 2022). It is not surprising that dysfunction of these organelles, crucial for cellular energy production, is associated with broad‐ranging disruption of ATP‐consuming metabolic processes. The potential short‐ and long‐term consequences to insect fitness and their relation to exposure time and repellent concentration cannot be elucidated from our study, but are likely to impact various physiological processes to some extent, including development, flight and response to environmental stressors (Jiao & Palli, 2022; Menail et al., 2022; Sargent et al., 2021; Treidel et al., 2023).

Our study yielded several notable findings related to the molecular responses associated with exposure to DEET and citronella in their vapour phase. The vast majority of olfactory genes that responded to repellent exposure had minimal differences in terms of fold change relative to the control, and this was particularly true for the antennae. We suspect that many of these significant genes are false positives, but is it certainly plausible that some could contribute to the repellency effect. The OR co‐receptor (ORCO) is essential for DEET repellency in mosquitoes (DeGennaro et al., 2013; Xu et al., 2014), but was only induced in the antennae of *Ae. aegypti* exposed to citronella. However, it appears to be constitutively expressed at high levels in chemosensory tissues, with overall read counts in the top 0.2% in the antennae and 21% in the mouthparts of control mosquitoes. Similarly, DEET did not activate ORCO‐expressing ORNs of *An. coluzzii*, regardless of concentration (Afify et al., 2019). In addition, Or27 (AAEL010418) was the most suppressed olfactory gene in the antennae in response to DEET. It has been shown to be significantly upregulated in *Ae. aegypti* after blood feeding, suggesting a role in host‐seeking behaviour (Ni et al., 2022). Notable for citronella exposure was the suppression of Or8, which is known to be expressed at high levels in the mouthparts of different mosquito species and plays a conserved role in sensing (R)‐1‐octen‐3‐ol (for review, see Speth et al. (2021)). Finally, three OBPs and one GR were differentially expressed in the bodies. Although this may seem counterintuitive, OBPs are found beyond chemosensory tissues (e.g. reproductive organs and pheromone glands), and the primary taste organs used by mosquitoes to sample host chemicals are the tarsal segments of the forelegs and midlegs (Rihani et al., 2021; Montell, 2025).

It must be emphasized that this research is unable to resolve whether any of the identified gene expression changes are behaviourally relevant molecular targets of DEET or citronella and thus truly connected to the mode of repellent action. P450 inhibition has been put forward as an explanation for DEET's ‘confusant’ hypothesis (Jaramillo Ramirez et al., 2012; Koloski et al., 2019), as well as repellency responses rooted in disrupting the metabotropic pathway of odour reception (Hansson et al., 2010; Jaramillo Ramirez et al., 2012; Wicher et al., 2008). However, it is conceivable that our experimental design did not capture the precise exposure and post‐exposure times necessary to truly inform on the molecular targets. We implemented a relatively long exposure to each repellent (15 min) in a closed system, followed by a 20 min post‐exposure treatment prior to examining changes in transcript accumulation. Our current understanding of the mode of action for volatile insect repellents is that the avoidance response is a rapid, often acute, physiological and biochemical phenomenon (a) (DeGennaro, 2015; Meier et al., 2025). As the trigger(s) for the repellency are presumably far removed from the time that gene expression was measured, potential associations between transcript abundance and repellency may be ambiguous. Case in point, AaTRP1a (AAEL001268) expression was not impacted by citronella exposure; however, it is directly activated by citronellal in *An*. g*ambiae*, and plays a role in citronellal‐induced repellency for other insects (Kwon et al., 2010; Shimomura et al., 2022). Further discrimination of the possible mechanisms of gene regulation related to mode of action could potentially be achieved through other techniques, such as single cell sequencing and DREAM (Koerte et al., 2018).

There are several other considerations related to our experimental design and methodologies that warrant consideration. In line with the aforementioned exposure times, citronella has considerably higher vapour pressure than DEET; thus, it evaporates more quickly and provides a shorter duration of protection (DeGennaro, 2015; Kaura et al., 2021). Nonetheless, our exposure period was well within the range for optimal protection by both repellents (Kongkaew et al., 2011; Yoon et al., 2015). We opted for higher concentrations to potentially elicit a more robust transcriptional response, as the Centers for Disease Control and Prevention (CDC) notes that DEET efficacy tends to peak at a concentration of ~50%. However, some studies indicate it plateaus at ~30%, and formulations of citronella >10% can cause skin sensitivity (Health Canada, 2002, Koren et al., 2003, Maia & Moore, 2011, Fradin, 2019, Koloski et al., 2020). In *Drosophila*, behavioural responses to DEET are highly sensitive to concentration, activating distinct, dose‐dependent sensory pathways and aversive behaviours (Guo et al., 2019; Shrestha & Lee, 2021). It is therefore conceivable that the observed transcriptional responses could be impacted by increasing/decreasing repellent concentration. Moreover, since the vast majority of differential expression was identified in the mouthparts, sequencing the maxillary palps and proboscis individually may have been beneficial; however, we encountered issues with library preparation using this approach. In addition, our read mapping was done at the gene‐level and *Ae. aegypti* is known to utilize alternative splicing to produce diverse transcripts from a single gene (Salvemini et al., 2013). However, this is not well characterized throughout the mosquito genome, and we would not be able to resolve reads mapping to conserved regions in multiple transcripts. In terms of the behavioural assays, both repellents are known to effectively deter host‐seeking behaviours, and this was consistent with our findings. Nevertheless, it is important to acknowledge that these assays were performed using membrane feeders in the absence of a human host or associated odours.

In conclusion, DEET and citronella exposure induced considerable molecular changes to the chemosensory tissues of *Ae. aegypti*. We identified molecular responses that could impact mosquito behaviours (e.g. host‐seeking), including CO_2_‐sensing gustatory receptors. These repellents also appear to disrupt cellular processes (e.g. mitochondrial function, metabolism) integral to insect physiology. Although any associations of our findings with repellent mode of action are presently unclear, this research provides a framework to better understand the precise roles of chemosensory tissues in xenobiotic responses. Future studies aimed at knocking down the expression of key molecular targets (e.g. RNAi, CRISPR) and evaluating the impact to host‐seeking behaviours of *Ae. aegypti* would better infer their functional relevance to DEET or citronella oil repellence. Moreover, examining global gene expression profiles after shorter exposure and post‐exposure times may provide additional insights into the repellency effect. It must also be emphasized that the olfactory systems of different mosquito genera may respond differently to repellent odours (Afify et al., 2019; Riffell, 2019). Culicinae (*Aedes* and *Culex*) diverged from Anophelinae (*Anopheles*) between 145 and 2000 mya (Moreno et al., 2010; Sieglaff et al., 2009), and *Ae. aegypti* and *An. gambiae* ORs share only 20% sequence homology (Zufall & Munger, 2016). Even different strains of *Ae. aegypti* show dissimilarities in their attraction rates towards human hosts and divergent expression profiles of olfaction‐related antennal genes (Mitra et al., 2021).

## AUTHOR CONTRIBUTIONS

**Ivan Drahun:** Conceptualization (equal); investigation (supporting); methodology (supporting); visualization (lead); writing – review and editing (supporting). **Jessica M. Sparrow:** Investigation (lead); writing – review and editing (supporting). **Cody W. Koloski:** Formal analysis (supporting); methodology (supporting); software (supporting); writing – review and editing (supporting). **Bryan J. Cassone:** Conceptualization (lead); data curation (lead); formal analysis (supporting); funding acquisition (lead); investigation (supporting); project administration (lead); resources (lead); software (lead); supervision (lead); validation (lead); writing – original draft (lead).

## FUNDING INFORMATION

This research was funded by the Natural Sciences and Engineering Research Council of Canada (NSERC) Discovery grant awarded to Bryan Cassone (RGPIN‐2023‐04126).

## CONFLICT OF INTEREST STATEMENT

The authors declare no conflict of interest.

## Supporting information


**Figure S1.** Illustration showing the chemosensory tissues and proteins involved in mosquito olfaction.

**Figure S2.** Visualization of significant genes shared among tissue types. (A) upregulated and (B) downregulated in response to DEET, and (C) upregulated and (B) downregulated in response to citronella. All contrasts are relative to the control (ethanol‐exposed). A = antennae; M = mouthparts; B = bodies.

**Table S1.** Primer sequences of genes subjected to quantitative real‐time PCR validation of differential expression (mouthparts) between control and DEET‐treated samples.

**Table S2.** Mapping statistics for sequenced cDNA libraries. Treatment: C = control; D – DEET; C = citronella. Tissue: A = antennae; B = bodies; M = mouthparts. The number associated with each sequencing library name denotes the technical replicate.

**Table S3.** Raw read counts for control (C), DEET‐treated (D) and citronella‐treated (Cr) samples derive from the antennae.

**Table S4.** Significant DAVID annotation clusters identified in *Aedes aegypti* female mouthparts and bodies in response to volatile DEET and citronella oil. The enrichment score and number of significant genes are also displayed for each pathway/treatment/tissue. No significant KEGG pathways were identified in the antennae. Red = downregulated in the repellent treatment in comparison to the control; Green = upregulated in the repellent treatment in comparison to the control.

## Data Availability

The raw sequence reads can be retrieved from the NCBI short sequence read archive (SRA) under the accession number PRJNA793033.https://www.ncbi.nlm.nih.gov/sra.
