# Supplementary material for: Transcriptional responses of Aedes aegypti chemosensory tissues in response to volatile DEET and citronella oil
Source: Insect Mol Biol. 2026 Jun 4;35(5):526–40. doi: 10.1111/imb.70049 (PMC13551536; doi:10.1111/imb.70049)
Supplement: Supplementary file 2 [file IMB-35-526-s003.docx]

**Table S1.** Primer sequences of genes subjected to quantitative real-time PCR validation of differential expression (mouthparts) between control and DEET-treated samples.

| **Gene ID** | **Primer Sequences** |
| --- | --- |
| AAEL002167 | F-CTGAAGGTGGACCATCCCAC |
|  | R-GCTCACGATAGCCGGATTCA |
| AAEL010058 | F-GTTGGTTCAGGTCATCCCGT |
|  | R-AGCCCTTTGGAAATCCTCCG |
| AAEL007603 | F-AAGAAGCTTCGCAACGGAGA |
|  | R-AGCTTGCCCAGTAACGACAA |
| AAEL006109 | F-TGTAACGATGCCAACGGTGA |
|  | R-TCCACTGGGTTCATCATGCC |
