# Supplementary material for: Transcriptional responses of Aedes aegypti chemosensory tissues in response to volatile DEET and citronella oil
Source: Insect Mol Biol. 2026 Jun 4;35(5):526–40. doi: 10.1111/imb.70049 (PMC13551536; doi:10.1111/imb.70049)
Supplement: Supplementary file 3 [file IMB-35-526-s004.docx]

**Fig. S2.** Visualization of significant genes shared among tissue types. (A) upregulated and (B) downregulated in response to DEET, and (C) upregulated and (B) downregulated in response to citronella. All contrasts are relative to the control (ethanol exposed). A = Antennae; M = Mouthparts; B = bodies.
