# Supplementary material for: Transcriptional responses of Aedes aegypti chemosensory tissues in response to volatile DEET and citronella oil
Source: Insect Mol Biol. 2026 Jun 4;35(5):526–40. doi: 10.1111/imb.70049 (PMC13551536; doi:10.1111/imb.70049)
Supplement: Supplementary file 4 [file IMB-35-526-s005.docx]

**Table S2.** Mapping statistics for sequenced cDNA libraries. Treatment: C = control; D – DEET; C = citronella. Tissue: A = antennae; B = bodies; M = mouthparts. The number associated with each sequencing library name denotes technical replicate.

| **Name** | **Number of reads (single)** | **Number of trimmed reads** | **%** | **Avg. length after trim** | **Reads mapped in pairs** | **%** |
| --- | --- | --- | --- | --- | --- | --- |
| C-A-1 | 84,909,498 | 84,873,001 | 99.96 | 90.46 | 54,291,678 | 64 |
| C-A-2 | 90,990,584 | 90,947,928 | 99.95 | 92.41 | 56,660,010 | 62.33 |
| C-A-4 | 64,316,718 | 64,287,926 | 99.96 | 93.21 | 39,492,216 | 61.46 |
| C-A-6 | 47,772,044 | 47,722,163 | 99.9 | 91.43 | 31,342,622 | 65.74 |
| C-B-2 | 77,873,384 | 77,337,784 | 99.31 | 99.38 | 69,043,406 | 89.68 |
| C-B-4 | 94,894,960 | 94,362,321 | 99.44 | 99.45 | 84,477,722 | 89.87 |
| C-B-5 | 56,462,388 | 56,271,423 | 99.66 | 99.81 | 50,218,180 | 89.47 |
| C-B-6 | 90,388,498 | 89,778,979 | 99.33 | 99.37 | 80,549,294 | 90.19 |
| C-M-1 | 56,694,074 | 56,672,508 | 99.96 | 92.71 | 38,564,798 | 68.07 |
| C-M-2 | 89,820,568 | 89,786,738 | 99.96 | 91.79 | 66,436,464 | 74.02 |
| C-M-4 | 79,124,252 | 79,091,835 | 99.96 | 91.05 | 52,496,780 | 81.81 |
| C-M-6 | 80,814,904 | 80,787,808 | 99.97 | 91.32 | 56,203,622 | 69.59 |
| Cr-A-1 | 55,524,050 | 55,476,491 | 99.91 | 93.1 | 35,308,064 | 63.7 |
| Cr-A-2 | 60,250,538 | 60,221,006 | 99.95 | 91.61 | 40,336,728 | 67.01 |
| Cr-A-3 | 75,396,656 | 75,366,880 | 99.96 | 94.88 | 46,600,262 | 61.86 |
| Cr-B-1 | 76,562,374 | 75,598,299 | 98.74 | 99.18 | 67,618,306 | 90.37 |
| Cr-B-2 | 76,167,280 | 75,756,250 | 99.46 | 99.78 | 67,531,726 | 89.52 |
| Cr-B-3 | 69,702,536 | 69,371,601 | 99.53 | 99.75 | 61,786,798 | 89.4 |
| Cr-M-1 | 67,788,082 | 67,752,306 | 99.95 | 93.92 | 43,579,018 | 64.35 |
| Cr-M-2 | 93,250,364 | 93,212,466 | 99.96 | 93.18 | 64,273,984 | 68.98 |
| Cr-M-3 | 70,758,864 | 70,720,613 | 99.95 | 92.63 | 46,158,898 | 65.3 |
| D-A-2 | 71,992,270 | 71,969,752 | 99.97 | 93.57 | 43,671,890 | 60.7 |
| D-A-3 | 81,845,570 | 81,815,559 | 99.96 | 92.9 | 50,998,882 | 62.36 |
| D-A-4 | 69,164,338 | 69,141,806 | 99.97 | 94.46 | 41,439,494 | 59.95 |
| D-B-1 | 45,267,168 | 45,119,821 | 99.67 | 99.61 | 40,880,066 | 90.84 |
| D-B-2 | 59,232,704 | 59,019,650 | 99.64 | 99.74 | 53,163,690 | 90.35 |
| D-B-3 | 81,394,844 | 80,992,530 | 99.51 | 99.43 | 72,547,322 | 89.92 |
| D-B-5 | 93,840,710 | 93,338,097 | 99.46 | 99.53 | 82,642,248 | 88.89 |
| D-M-1 | 60,736,922 | 60,715,940 | 99.97 | 93.97 | 40,284,342 | 66.37 |
| D-M-2 | 71,784,780 | 71,753,237 | 99.96 | 92.18 | 48,063,994 | 67.01 |
| D-M-3 | 53,521,922 | 53,504,173 | 99.97 | 92.88 | 35,858,548 | 67.04 |
| D-M-4 | 70,568,836 | 70,544,246 | 99.97 | 91.1 | 49,047,364 | 69.55 |
