# Supplementary material for: Transcriptional responses of Aedes aegypti chemosensory tissues in response to volatile DEET and citronella oil
Source: Insect Mol Biol. 2026 Jun 4;35(5):526–40. doi: 10.1111/imb.70049 (PMC13551536; doi:10.1111/imb.70049)
Supplement: Supplementary file 5 [file IMB-35-526-s006.docx]

**Table S4.** Significant DAVID annotation clusters identified in *Aedes aegypti* female mouthparts and bodies in response to volatile DEET and citronella oil. The enrichment score and number of significant genes are also displayed for each pathway/treatment/tissue. No significant KEGG pathways were identified in the antennae. Red = downregulated in the repellent in comparison to the control; Green = upregulated in the repellent in comparison to the control.

|  | **Annotation Cluster** | **Enrichment Score** | **No. Genes** |
| --- | --- | --- | --- |
|  | Oxidative phosphorylation / electron transport | 26 | 110 |
|  | Tricarboxylic acid cycle | 13.31 | 44 |
|  | Translation/ribosome | 8.36 | 45 |
|  | Glycolysis | 6.07 | 15 |
|  | Carbohydrate binding | 4.53 | 13 |
|  | Proteolysis | 4.15 | 85 |
|  | Motor proteins / cytoskeleton | 3.2 | 21 |
|  | Lipoic acid metabolism | 3.16 | 11 |
|  | Innate immune response | 2.92 | 42 |
| **DEET** | FAD / electron carrier molecule | 2.83 | 21 |
|  | Protease inhibitor | 2.63 | 10 |
|  | Mitochondria import/transport | 2.27 | 10 |
|  | Iron-sulfur | 2.14 | 11 |
|  | ATP synthesis | 2.09 | 17 |
|  | Insect CytP450 Detox | 2.06 | 53 |
|  | Protein folding | 2.05 | 14 |
|  | Signal transduction / Cytoskeleton / Calcium | 1.91 | 29 |
|  | Actin / cytoskeleton | 1.73 | 29 |
|  | Cysteine and methionine metabolism | 1.71 | 15 |
|  | Thioredoxin | 1.38 | 14 |
|  | Transmembrane | 1.39 | 13 |
|  | Mitochondrion inner membrane | 49.57 | 102 |
|  | Oxidative phosphorylation | 40.77 | 97 |
|  | Electron transport | 29.95 | 50 |
|  | Tricarboxylic acid cycle | 9.71 | 26 |
| **Citronella** | Translation | 8.79 | 32 |
|  | Iron-sulfur | 4.3 | 12 |
|  | ATP synthesis | 3.09 | 14 |
|  | Motor proteins / cytoskeleton | 2.58 | 10 |
|  | Lipoic acid metabolism | 1.91 | 11 |
|  | Transmembrane | 1.86 | 125 |
|  | Transmembrane | 2.05 | 13 |
|  | Tricarboxylic acid cycle / Lipoic acid metabolism | 6.57 | 21 |
| **DEET** | Chitin binding | 1.96 | 13 |
|  | Transcription | 2.83 | 41 |
| **Citronella** | Transmembrane | 1.93 | 14 |
