# Supplementary material for: Transcriptional responses of Aedes aegypti chemosensory tissues in response to volatile DEET and citronella oil
Source: Insect Mol Biol. 2026 Jun 4;35(5):526–40. doi: 10.1111/imb.70049 (PMC13551536; doi:10.1111/imb.70049)
Supplement: Supplementary file 6 [file IMB-35-526-s002.docx]

**Fig. S1.** Illustration of the chemosensory tissues and proteins involved in mosquito olfaction.
